# The Crying Need for a Better Response Assessment in Rectal Cancer

**DOI:** 10.1007/s11864-023-01125-9

**Published:** 2023-09-13

**Authors:** Samuel Amintas, Nicolas Giraud, Benjamin Fernandez, Charles Dupin, Quentin Denost, Aurelie Garant, Nora Frulio, Denis Smith, Anne Rullier, Eric Rullier, Te Vuong, Sandrine Dabernat, Véronique Vendrely

**Affiliations:** 1 Tumor Biology and Tumor Bank Laboratory, CHU Bordeaux, F-33600 Pessac, France; 2 BRIC (BoRdeaux Institute of onCology), UMR1312, INSERM, University of Bordeaux, F-33000 Bordeaux, France; 3 Department of Radiation Oncology, CHU Bordeaux, F-33000 Bordeaux, France; 4 Surgery Department, CHU Bordeaux, F-33600 Pessac, France; 5 Bordeaux Colorectal Institute, F-33000 Bordeaux, France; 6 UT Southwestern Department of Radiation Oncology, Dallas, USA; 7 Radiology Department, CHU Bordeaux, F-33600 Pessac, France; 8 Department of Digestive Oncology, CHU Bordeaux, F-33600 Pessac, France; 9 Histology Department, CHU Bordeaux, F-33000 Bordeaux, France; 10 Department of Radiation Oncology, McGill University, Jewish General Hospital, Montreal, Canada; 11 Biochemistry Department, CHU Bordeaux, F-33000 Bordeaux, France

**Keywords:** Rectal cancer, Chemoradiotherapy, Non-operative management, Rectal preservation, Response assessment, TRG, Rectal brachytherapy

## Abstract

Since total neoadjuvant treatment achieves almost 30% pathologic complete response, organ preservation has been increasingly debated for good responders after neoadjuvant treatment for patients diagnosed with rectal cancer. Two organ preservation strategies are available: a *watch and wait* strategy and a local excision strategy including patients with a near clinical complete response. A major issue is the selection of patients according to the initial tumor staging or the response assessment. Despite modern imaging improvement, identifying complete response remains challenging. A better selection could be possible by radiomics analyses, exploiting numerous image features to feed data characterization algorithms. The subsequent step is to include baseline and/or pre-therapeutic MRI, PET-CT, and CT radiomics added to the patients’ clinicopathological data, inside machine learning (ML) prediction models, with predictive or prognostic purposes. These models could be further improved by the addition of new biomarkers such as circulating tumor biomarkers, molecular profiling, or pathological immune biomarkers.

**Graphical Abstract:**

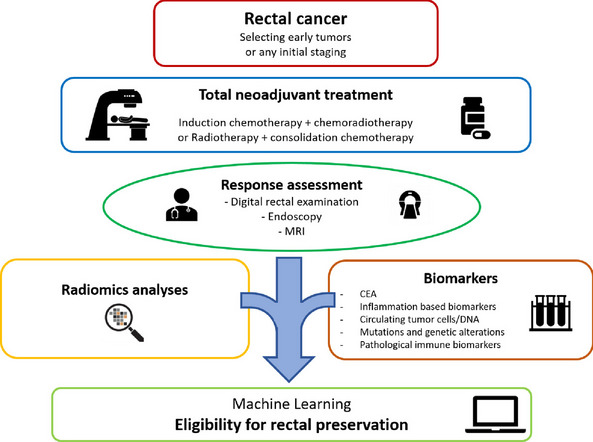

## Introduction: in need of an accurate chemoradiotherapy response assessment

The management of rectal cancers has deeply evolved over the past decades toward a multidisciplinary strategy, combining radiotherapy, chemotherapy, and surgery. Local recurrence rates, from 20 to 40% depending on the initial staging before 1990, have drastically dropped to less than 10% with pre-operative radiotherapy and the standardization of total mesorectal excision (TME) described by Heald et al. in 1986 [1, 2]. Standard treatment included chemoradiotherapy followed by surgery and sometimes adjuvant chemotherapy [3–5]. Recently, PRODIGE23 and RAPIDO trials demonstrated improvement of disease-free survival by placing chemotherapy before or after radiotherapy in a total neoadjuvant treatment (TNT) [6, 7]. However, this strategy, considered to be optimal regarding oncological outcomes, is not toll-free, with a high morbidity rate of around 50%, including 20% of pelvic infections (abscesses and anastomotic fistulas), 10% of occlusions, and 20% of medical complications [8]. Finally, half of the patients have functional sequelae such as digestive disorders (diarrhea, constipation, anal incontinence) or genitourinary disorders (impotence, anejaculation, urinary incontinence) [9]. Only 15% of patients display sterilized tumors after chemoradiotherapy (CRT) for T3/T4 rectal cancer and are eligible for organ preservation with decreased surgical morbidity and increased quality of life [10, 11]. Total neoadjuvant strategy doubles this number, rendering organ preservation possible for one-third of the patients [6, 7]. Organ preservation has been increasingly debated for good responders after CRT in recent years, with encouraging results but still many issues [12, 13]. Two organ preservation strategies are available: a *watch and wait* strategy and a local excision (LE) strategy including patients with a near clinical complete response [11]. A major issue is the selection of patients according to the initial tumor staging or the response assessment. Despite modern imaging improvement, identifying complete response remains challenging. The main advantage of local excision is to provide a precise evaluation of tumor response with gold standard histopathology, while the watch-and-wait option relies on less accurate clinical and radiological evaluations. Moreover, treatment strategy may be adapted according to the pathologic tumor response after local excision. Indeed, bad pathological tumor responders will receive a completion TME, at the cost of increased morbidity. However, a near-clinical complete response assessed by LE could have become a clinical complete response a few weeks or months later, as seen with the watch-and-wait strategy. Indeed, watch and wait is not based on evaluation at a given time, but rather on repeated evaluations over time by attentive surveillance. The main drawback is the risk of persistence of imaging-undetectable residual tumor cells requiring radical surgery in one third of the patients [14]. Although “oncologically safe,” salvage surgery reaching 90% of R0 resections compromises quality of life with more than 50% abdominoperineal excision and definitive colostomy [15]. Moreover, even if local regrowth could be treated safely with salvage surgery, the risk of distant metastases persists, possibly aggravated by the uncontrolled primary tumor as a cause of dissemination [16].

Regardless of the chosen strategy, local excision or watch and wait, the gastrointestinal oncology community needs an accurate tumor response assessment approach. This review details the current selection of patients eligible for organ preservation and identifies the perspectives for better response assessment of CRT.

## The strategies for rectal preservation

### The selective strategy

**Fig. 1:**
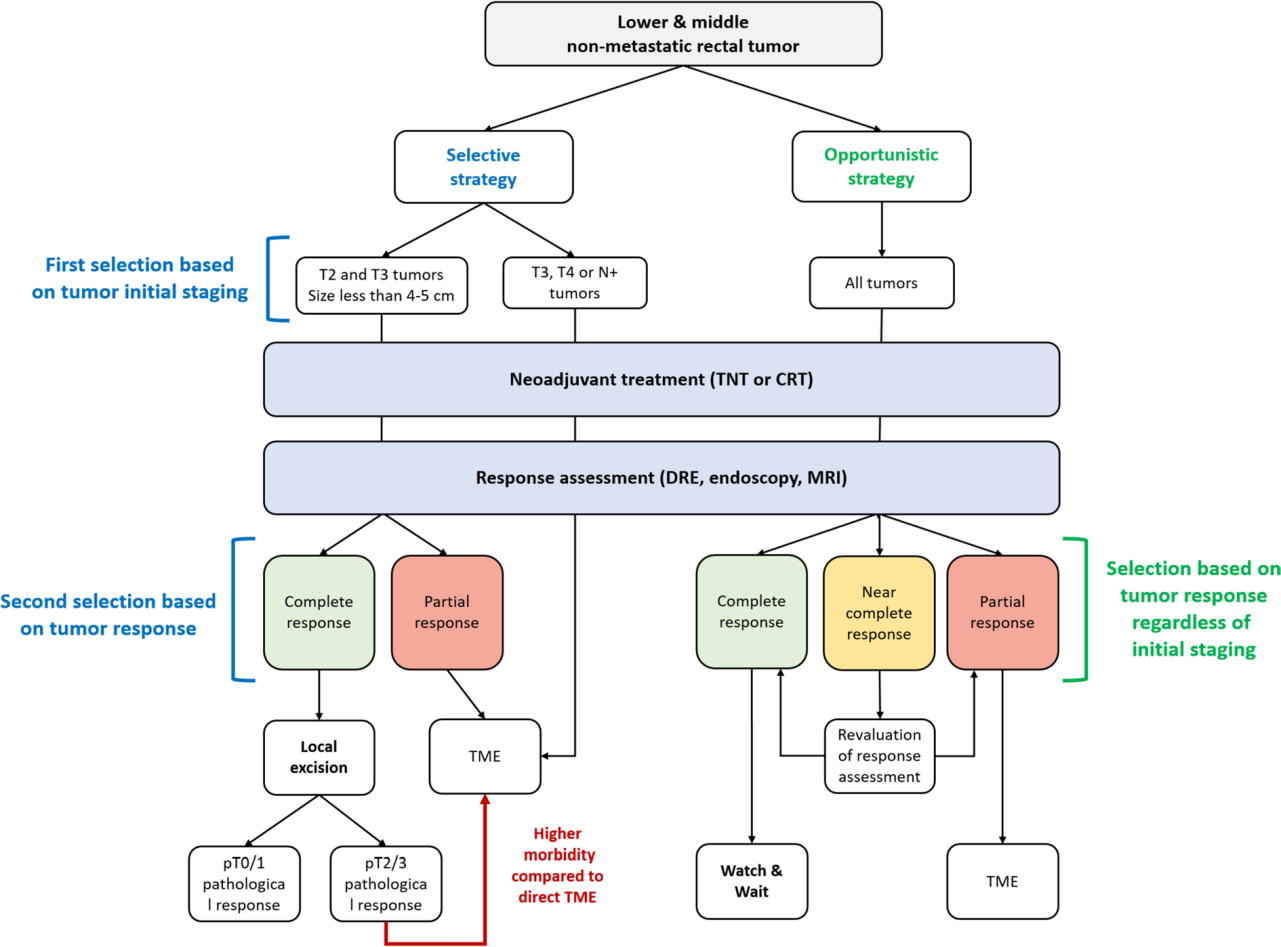
Selective and opportunistic strategies for selection of patients eligible to organ preservation. TNT, total neoadjuvant treatment; CRT, chemoradiotherapy; DRE, digital rectal examination; MRI, magnetic resonance imaging; TME, total mésorectum excision

Selecting patients according to initial staging, including small tumors T2 or T3 less than 4–5 cm, was a common hypothesis, tested in most LE studies, and confirmed by the high rates of near-complete or complete pathological response (50 to 80%) [14]. A major concern regarding rectal preservation is the risk of leaving invaded nodes in the mesorectum. GRECCAR 2 trial, the first randomized trial comparing LE and TME in good responders after chemoradiotherapy, showed that this risk was low in patients with a good pathological response and small tumors at initial staging. Indeed, no pathological node was found in patients with pT0-1 in the TME group versus 8% in patients with pT2 tumors and 40% for pT3 tumors [17]. Moreover, the recently published 5-year follow-up confirmed the oncological safety of the strategy [18••]. No difference was found in terms of overall survival (84% [19–31, 32, 33–37] vs 82% [19–31, 32, 33–36, 38, 39]; 0·92 [0·38–2·22]; *p* = 0·85), disease-free survival (70% [19–25, 38–52] vs 72% [19–28, 38, 39, 42–52]; 0·87 [0·44–1·72]; *p* = 0·68), or cancer-specific mortality (7% [3–17] vs 10% [5–17, 18••, 53, 54]; 0·65 [0·17–2·49]; *p* = 0·53) between the LE and TME groups. This trial also showed that LE followed by TME in pT2 and pT3 patients, justified by the risk of mesorectal lymph node invasion, was more morbid than direct TME with similar tumor stages (Fig. 1). At the same time, patients who underwent local excision only of pT0/T1 tumors showed a better quality of life compared patients undergoing TME for the same pT0/T1 stage Thus, although pathological response provides the “true response,” LE is not the best option to access this critical information for incomplete responders who require additional surgery. GRECCAR2 results clearly show that accurate CRT response is needed to choose the best surgical option or no surgery at all.

### The opportunistic strategy

*Watch and wait* studies have included patients with more advanced tumors at initial staging, but with clinical complete response. As stated by the systematic review and meta-analysis of 23 studies and 867 patients, pooled 2-year local regrowth was 15.7% (*95%CI* 11.8–20.1), and no significant difference was found in terms of cancer-specific mortality or overall survival between patients managed with the *watch and wait* strategy as compared to patients with clinical complete response treated by surgery [53]. Interestingly, 67% of tumors were initially staged as T3 and 52% were initially node-positive, with no differences for patients treated by surgery or watch and wait after a clinical complete response. As patients in clinical complete response after chemoradiation were selected regardless of the initial tumor staging, this suggested that tumor response was more relevant than initial tumor staging [54]. Individual participant data pooled analysis of risk factors for recurrence after neoadjuvant radiotherapy and transanal excision confirmed that post-treatment staging, i.e., “true” pathological response, predicted overall survival better than initial staging [14]. Again, the evaluation of the “true” response is critical and should be available without any kind of tumor excision, for complete organ preservation.

Thus, a good response assessment is mandatory for rectal preservation, regardless of the strategy.

## Assessing the response

### Defining clinical complete response

Clinical complete response has been usually defined as “no tumor felt, no tumor seen” using digital rectal examination and proctoscopy. The Brazilian team added criteria in favor of clinical complete response such as whitening of the mucosa, telangiectasia, or fibrosis whereas persisting ulcer and stenosis might reflect incomplete response [55].

However, clinical complete response is not always associated with pathologic complete response, as stated by Bujko et al. with 37.5% of samples with residual tumor at pathology whereas complete response was assessed clinically [56]. Moreover, pCR was found in 33% of patients considered partial responders by clinical assessment.

### Improving good responder selection by imaging

Several studies have looked at the contribution of recent imaging progress such as magnetic resonance imaging (MRI) and positron emission tomography (PET) in the definition of the complete response after chemoradiotherapy. In particular, post-treatment MRI was promising since tumor regression grade (mTRG) and tumor stage after chemoradiotherapy were correlated with the histopathological response, unlike the Response Evaluation Criteria In Solid Tumors [RECIST] method [57]. Similarly, the study of diffusion sequences increased the sensitivity of MRI in complete response assessment from 40 to 52%. The specificity was good, between 89 and 98% [58]. As for the PET scan, the *Habr-Gama* team showed that a variation in the standard uptake value (SUV) greater than 76% between the initial PET and that at 12 weeks after chemoradiotherapy was significantly associated with the clinical complete response. However, they considered PET scan less reliable (85% of well-classified cases) than clinical evaluation (91% of well-classified cases) [59]. Other teams suggest that early variation of the SUV is highly predictive of the complete response [60]. Finally, joint analysis of the data from the published series showed that PET scan and diffusion-weighted MRI distinguished nonresponsive from responsive tumors but remained imprecise in the identification of complete responders [61]. Indeed, in the ESCP cohort, of the 2572 patients undergoing rectal cancer surgery in 277 participating centers across 44 European countries, 673 (26.2%) underwent chemoradiotherapy and surgery [62]. The pCR rate was 10.3% (67/649), with a partial response in 35.9% (233/649) patients. Comparison of AJCC stage determined by post-treatment yMRI with final pathology showed understaging in 13% (55/429) and overstaging in 34% (148/429). Agreement between yMRI and final pathology for T-stage, N-stage, or AJCC status were each graded as “fair” only (*n* = 429, Kappa 0.25, 0.26, and 0.35, respectively) [62]. In fact, reliable response assessment is still limited to expert centers, which included small series, with the need for validation by larger prospective studies. In addition, the question of the optimal time for response assessment remains debated: an early evaluation might underestimate a complete response. Thus, the minimum time for assessment recommended by the Brazilian team increased from 6 to 8 weeks and finally to 12 weeks in their most recent studies [55].

## Near-future solutions to improve response assessment

### Biomarkers

Research and study of biological markers predicting CRT tumor response and classifying good and bad responders, whether they are of cellular or molecular nature, constitute an active field of research (Table 1).

**Table 1:** Main biomarkers evaluated for response assessment after chemoradiotherapy

Responses assessment method *(reference)*			Main performances for nCRT tumor response prediction	Limits and needed developments for clinical use
Tumor biomarkers	Wild-type P53 status [42]		Good pathological response; *RR* = 1.20, *95% CI* = 1.01–1.43, *p* = 0.043	Lack of harmonized detection methods (immunohistochemistry or gene analysis)
			Complete pathological response; *RR* = 1.92, *95% CI* = 1.26–2.91, *p* = 0.002	
			Poor pathological response; *RR* = 0.91, *95% CI* = 0.68–1.12, *p* = 0.284	
	Positive MSI status [47]		Reduced pCR rate; *OR* = 0.65, *95% CI* 0.43–0.96	Lack of harmonized detection methods (immunohistochemistry or molecular analysis)
	Negative MSI status [46]		Association with pCR: *p* = 0.048	
Circulating biomarkers	Pre-nCRT elevated CEA plasma concentration[63]		Inverse correlation with pathologic complete response rate; *OR* = 2	Recommendations for a pre-nCRT CEA threshold
	Blood inflammation-based index	Elevated pre-nCRT platelets rate [63]	Association with lower pathologic complete response and good pathological response rate; *p* = 0.001	Conflicting studies Lack of homogenous cut-offs Need for larger prospective validation studies
		Elevated post-nCRT NLR [64]	Independent negative predictive factor for pathologic complete response; *OR* = 0.365, *95% CI* 0.145–0.918	
	ctDNA	Post-nCRT ctDNA drop [65]	Positive association between MAF decrease after nCRT and pCR; *p* = 0.015	Need for larger prospective validation studies Lack of ctDNA detection assays harmonization
		Post-nCRT ctDNA positive detection [66••]	Positive ctDNA status after nCRT was associated with mrTRG primary tumor response; *p* = 0.03	
	CTC	Low pre-nCRT CK20 + circulating cells count [67]	Positive association with response to nCRT; *p* = 0.03	Need for larger prospective validation studies Increasing CTC detection by enhancing technologies and technical tests
		Low post-nCRT CTC count [68]	Positive association with response to nCRT; *p* < 0.001	

*AUC* area under the curve, *CEA* carcinoembryonic antigen, *ctDNA* circulating tumor DNA, *CI* confidence interval, *CTC* circulating tumor cell, *MSI* microsatellite instability, *nCRT* neoadjuvant chemoradiotherapy, *NLR* neutrophils to lymphocytes ratio, *OR* odds ratio, *pCR* pathologic complete response, *RR* relative ratio, *mrTRG* MRI tumor regression grade

#### Circulating biomarkers

##### Circulating proteins and peptides

Carcinoembryonic antigen (CEA) is a widely recognized biomarker for prognosis and disease monitoring in colorectal cancer. Several studies showed that low pre-CRT CEA levels with various cut-off values were associated with a good tumor response or a pathologic complete response (pCR) [69, 70]. In addition, some studies showed that post-CRT CEA level was an independent predictor of tumor response [71]. A recent meta-analysis, including 32 publications, describes a significant inverse correlation between pre-CRT CEA level and pCR (OR 2.00) [63]. The authors recommend a cut-off value of serum CEA level between 3 and 5 ng/ml. Considering these interesting results, CEA is used for the follow-up and the early detection of metastatic evolution. However, its role in predicting CRT response remains minor given the absence of elevated circulating concentrations in localized rectal cancer. A retrospective study of 947 patients who received CRT, found elevated fibrinogen levels together with CEA before CRT, predictive of downstaging, primary tumor regression, and pCR [72].

##### Inflammation-based biomarkers

Inflammation markers can be predictive of neoadjuvant treatment efficiency in multiple tumors (reviewed in [73]). For instance, baseline thrombocytosis was inversely correlated to response to CRT in a retrospective study including 965 rectal cancers [74]. Pathologic complete response was significantly lower in patients with an elevated pre-CRT platelet count (12.8% vs 22.1%, *p* < 0.001). The combination of pre-CRT platelet and neutrophil counts also offered predictive value of CRT efficiency [75], and the impact of baseline leukocytosis (BL) has been confirmed in a randomized phase III clinical trial CAO/ARO/AIO‐04 including more than 1200 patients with a 50-month follow-up [76]. BL was an independent prognostic factor for disease‐free survival (*HR* 1.457; *95% CI* 1.163–1.825; *p* = 0.001), distant metastasis (*HR* 1.696; *95% CI* 1.266–2.273; *p* < 0.001), and overall survival (*HR* 1.716; *95% CI* 1.264–2.329; *p* = 0.001). Conversely, treatment‐induced leukopenia was correlated with a favorable DFS (*p* = 0.037), distant metastasis (*p* = 0.028), and OS (*p* = 0.012). In addition, neutrophil to lymphocyte ratio (NLR) was a negative predictive marker for CRT response independently associated with decreased RFS (*HR*: 2.3; *95% CI*, 1.06–4.98) [64]. These results were not confirmed in an independent study [77]. Therefore, the exact predictive value of inflammation-based markers needs further validation in larger studies.

##### Circulating tumor biomarkers

Circulating tumor cells (CTCs) have been found as promising monitoring biomarkers in different cancer types, including colorectal cancer [78]. Sun et al. compared CTC and CEA levels for predicting rectal cancer CRT response [79]. CTCs were present in all patients with higher counts in metastatic patients and were absent in healthy controls. CRT tumor response correlated with kinetics of both CTCs and CEA levels (pre-post CRT). Interestingly, CTCs kinetic was superior to CEA in treatment response prediction. In addition, CTC count decreased in good responders regardless the use of distinct analysis methods [67, 68, 80]. Counts of CTC expressing thymidylate synthase (TYMS), the main target of 5-FU, and RAD23 homolog B (RAD23B), a protein involved in double-strand break DNA repair, were undetectable after CRT [81]. Despite these positive results, one of the limitations of CTC use for routine prediction of CRT response remains the very low rate of cells present in the blood flow, especially for localized stages tumors [82]. This limitation could be overcome by exploring the clinical value of other circulating tumor elements such as circulating tumor DNA (ctDNA) and extracellular vesicles.

Circulating cell-free DNA (cfDNA) and circulating tumor DNA (ctDNA) show higher blood levels than CTCs and are more easily detected and quantified with current technologies. Multiple studies interrested in predicitve value of pre- and post-CRT cell-free DNA (cfDNA) blood levels. Even if pre-CRT cfDNA levels were not found significantly different between good and poor responders, the decrease of cfDNA levels at baseline compared to post-CRT was significantly higher for good responders than for poor responders [47, 48]. Tie et al. analyzed ctDNA from 154 rectal cancer patients before CRT, after CRT, and after surgery [85]. ctDNA was detectable in 77%, 8.3%, and 12% of pre-CRT, post-CRT, and post-surgery plasma samples, respectively. Disappointingly, no association between post-CRT ctDNA status and pCR was found. The same conclusion was drawn for the conversion of ctDNA status from positive at baseline to negative at 4–6 weeks after completing CRT (pCR vs non-pCR, 95% vs 88%, *p* = 0.46). Recently, ctDNA levels were assessed before and after TNT for 144 paired plasma samples of 72 patients [85]. ctDNA was detected in 83% of samples before TNT and 15% following TNT. Despite the absence of association between ctDNA status and pathological tumor response, detectable pre-surgery ctDNA was associated with systemic recurrence, shorter DFS (*HR*, 4; *p* = 0.033), and shorter OS (*HR*, 23; *p* < 0.0001). Another study reported a significant association between decrease of ctDNA mutant allele frequency after nCRT and pCR (≥ 80% vs < 80%, *p* = 0.015) [65]. Moreover, a positive association was found between ctDNA positive detection after nCRT and metastatic recurrence in 3 independent studies [65, 66••, 86]. A recent meta-analysis highlight post-operative ctDNA as the most predictive prognostic factor of all investigated time points of treatment [87]. Therefore, circulating tumor DNA analysis appears to be the preferred liquid biopsy strategy for response assessment to CRT and could be an important asset for therapy adjustment and patient follow-up. Recently, key recommendations for ctDNA application and integration in rectal cancer management were published, including standardization of sample collection and use of high sensitivity assays [88]. Moreover, authors stressed out the need for more neoadjuvant clinical trials testing ctDNA positivity at diagnosis as a prognosis factor and changes in ctDNA as a treatment response biomarker. In line with this missing data, ctDNA analysis in *watch and wait* cohorts is still missing.

Next to CTCs and ctDNA, study of extracellular vesicles as potential biomarkers represent a research field of growing interest. Few studies exploring differential molecular content of extracellular vesicles have been recently published or are ongoing (NCT04852653; [89]). This aspect of liquid biopsy deserves close attention, as preliminary data seem promising.

#### Tumor characterization

##### Mutations and genetic alterations

The relationship between *TP53* status and response to CRT has been extensively studied with conflicting results [40, 90, 91]. A meta-analysis of 30 studies highlighted a correlation between wild-type *TP53* status and good response to CRT in 1830 rectal cancer patients with risk ratios (*RR*) of 1.30 (*p* < 0.001), 1.65 (*p* = 0.003), and 0.85 (*p* = 0. 007) for good, complete, and poor response, respectively [41]. In a recent study analyzing *KRAS*, *BRAF*, *NRAS*, *PIK3CA*, and *TP53* gene mutations in 210 rectal tumors, only *TP53* mutation was associated with poor pathological tumor regression (23% vs 36%, *p* = 0.05) [42]. The presence of *KRAS* gene exon 2 activating mutations (codons 12 and 13) have also been described as an independent predictive of poor response to CRT (odds ratio = 0.34, *p* < 0.01) [43].

Along with *TP53* pathway, the mismatch repair (MMR) system contributes to genomic stability. Studies have sought to assess the prognostic and predictive role of tumor microsatellite instability (MSI) status on CRT response. In a cohort of 1103 patients with curatively resected stage II/III rectal cancer, MSI positive status did not correlate with DFS (*HR* = 1; *p* = 0.994) or OS (*HR* = 0.85; *p* = 0.778) [44]. It should be noted that the rate of positive MSI tumors was only about 2.2%, which is below the rates traditionally described in the literature (10–15% depending on studies). By contrast, 2 recent studies assessed a negative predictive role of MSI positive status for tumor response to CRT. MSI status assessment was performed by PCR-based analysis or by immunohistochemistry assay. While one study found MSI negative (pMMR) status as significantly correlated with pCR (*p* = 0.048) [45], the other reported that all cases with pCR were pMMR, however without obtaining significant statistical value [46]. Those results were confirmed by Hasan et al. who highlighted an independent association between the MSI positive status and the reduction in pCR after chemoradiotherapy (*OR* = 0.65) in a cohort of 5086 advanced rectal cancer patients [47]. Those 3 last studies present classical MSI positive rates with 12%, 10.8%, and 13.4% of tumors. However, it is likely that immunotherapy based neoadjuvant treatments will gradually replace radiochemotherapy for MSI rectal tumors as major pCR rates have been recently described [48, 49]. However, immunological profiling of pMMR/MSS rectal tumors may also contribute to the selection of patients eligible for an organ preservation strategy.

##### Pathological immune biomarkers

The development of immunotherapy over the last 10 years has led to the emergence of numerous predictive immune biomarkers like PD1 and PDL-1 expression, mutational tumor burden (TMB), and tumor-infiltrating lymphocytes (TIL). These markers could be more relevant if combined. Indeed, the Immunoscore combining total tumor-infiltrating T cell counts and cytotoxic tumor-infiltrating T cell counts was predictive of CRC prognosis [50, 51]. Interestingly, a diagnostic biopsy adapted Immunoscore (ISB) was proven efficient for predicting response to CRT and better identifying the patients eligible for an organ preservation strategy [52]. The authors found a positive association between ISB and post-CRT histologic response (*p* < 0.001). High ISB identified patients at lower risk of relapse or death compared with low ISB (*HR*, 0.21; 95% confidence interval (CI), 0.06–0.78; *p* = 0.009). This performance was confirmed for DFS in a validation cohort. Moreover, ISB was an independent parameter, more informative than pre- (*p* < 0.001) and post-CRT (*p* < 0.05) imaging to predict DFS. By combining post-CRT ISB and imaging, the authors discriminated very good responders.

### Radiomics and AI

**Fig. 2:**
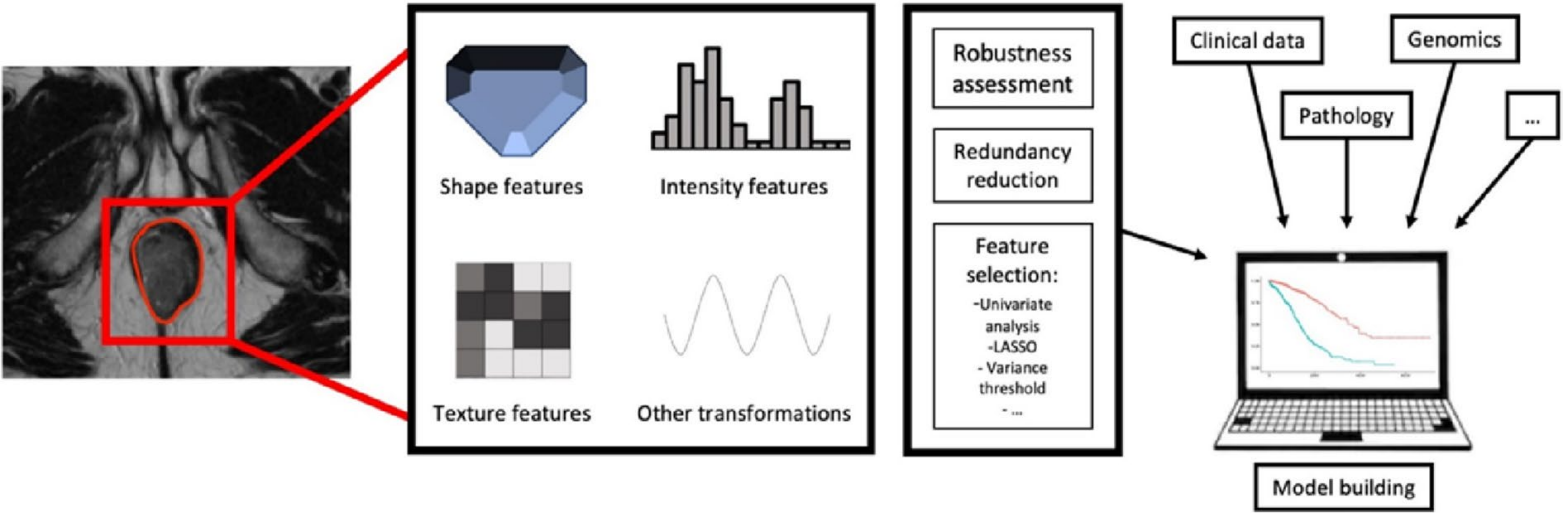
Radiomics workflow and integration in ML model building

Radiomics is the noninvasive extraction of quantitative high-dimensional features from morphological and functional imaging, providing novel imaging biomarkers aiming to unravel imaging patterns and characteristics beyond visual inspection alone [38]. Promising results in oncology are emerging for a wide range of cancers [19, 39]. After obtaining these indicators, the subsequent step is to include them inside machine learning (ML) prediction models, for example, with predictive or prognostic purposes (Fig. 2). ML is a subset of artificial intelligence in which an algorithm, supervised with labeled data or unsupervised, learns by pattern recognition and inference from a dataset encompassing a large number of variables [20, 21]. It then issues predictions on a testing set, which are compared to the actual outcome to assess the model’s performance. ML algorithms currently mainly include logistic regression, random forests, or support vector machines methods.

For rectal cancers, these models have been tested to predict initial tumor grading, genetic profile, or lymph node status, with auspicious performances [22, 23]. They could also be of paramount interest with regard to personalized medicine, notably in organ preservation strategies, by helping to foretell the pathologic complete response after the neoadjuvant sequence [24]. The contribution of baseline and/or post-treatment MRI, PET-CT, and CT radiomics in such models, alongside patients’ clinicopathological data, has been abundantly reported with positive results [23, 25–31]. For example, Liu et al. built a logistic regression model learning with 152 patients comprising both (pre- and post-CRT) radiomic and independent clinicopathological risk factors to predict pCR after CRT, obtaining an AUROC of 0.976 in the validation cohort (*n* = 70) [32]. Delta-radiomics, a measure of the evolution of quantitative radiomic features during and after the treatment [28], was explored more recently and can be fueled by routine positioning verification images performed during the radiation treatment. While most current accelerators carry low-resolution cone beam CT, the new MRI-guided linacs could provide data amenable to radiomic analysis without increasing the radiation exposure [30].

Reproducibility of radiomic feature extraction was evaluated for rectal cancers for both MRI and CT and highlighted several adequately repeatable features, thus supporting the use of radiomics for these malignancies [33–35]. However, the lack of standardized imaging procedures, radiomic extraction methods, and ML model building approaches are currently preventing radiomic tools to be translated into clinical practice. As most published results are based on mono- or pauci-centric retrospective data, there is a glaring need for prospective large sample multicentric studies and external verification. Another potential evolution in radiomics could also be the development of neural network-based deep learning (DL) models, able to learn directly from raw images without requiring image segmentation and intermediate feature extraction [36]. While possibly advantageous in terms of reproducibility and repeatability, studies to date using DL-based radiomics remain preliminary [37, 92].

Finally, as ML typically thrive with high-dimensional data, adding for instance clinicopathological or biological data usually outperforms models based on radiomic features alone. This is also why the research of new biomarkers is of critical interest to further improve the predictive performances of these methods.

## Conclusion

Response assessment has become a key point to enable personalized therapeutic strategies for each patient with rectal cancer: it has augmented clinicians’ ability to identify those eligible for organ preservation. Current clinical and radiological assessments lack efficiency. Promising tools integrating radiomics analyses and molecular biomarkers into machine learning algorithms could be a game-changer. Accurate estimation of complete response and risk of local/general recurrence while balancing the risk of functional sequelae could help the physician in the decision process, taking into account the patient’s preference.
